# A new image encryption scheme based on coupling map lattices with mixed multi-chaos

**DOI:** 10.1038/s41598-020-66486-9

**Published:** 2020-06-17

**Authors:** Xingyuan Wang, Nana Guan, Hongyu Zhao, Siwei Wang, Yingqian Zhang

**Affiliations:** 1 School of Information Science and Technology, Dalian Maritime University, Dalian, 116026 China; 2 Faculty of Electronic Information and Electrical Engineering, Dalian University of Technology, Dalian, 116024 China; 3 School of Information Science and Technology, Xiamen University Tan Kah Kee College, Fujian, 363105 China

**Keywords:** Information technology, Nonlinear optics

## Abstract

As a kind of spatiotemporal chaos, coupled map lattice (CML) is widely applied into image encryption because of its advantages of more complex dynamical behavior and lower computational overhead. Firstly, this paper proposed a novel spatiotemporal chaos model (MCML) by mixing Logistic, Sine and Tent maps into CML map together. Beyond that, we also change the structure of CML and the coupling method in different lattices. Bifurcation diagram, Lyapunov exponents and NIST test are employed to measure the chaotic behaviors of the MCML system. Secondly, by applying MCML chaos, we design a new key binding and distribution rule, the improved diffusion scheme to encrypt image. Furthermore, the novel bit Z-scan scrambling method also be used to enhance the security of the encryption scheme. Finally, a large number of experimental results prove that our proposed scheme is suitable for image encryption and has high security against common attacks.

## Introduction

Nowadays, with the rapid development of big data and mobile internet, media information especially image is gradually becoming the most important information carrier in social communication. Images are widely used in the fields of information exchange, business, personal privacy, military and so on, therefore, it occupies an increasing proportion in the network information space. Compared with the text, it has the characteristics of strong correlation between adjacent pixels, large amount of data and redundant information, so that the conventional encryption methods such as AES and DES have encountered severe challenges^1^. The application of new technology, the rapid enhancement of computing capacity and large data analysis, caused the current image encryption algorithms to emerge the hidden dangers in security. It’s necessary to study novel and safer cryptosystem to meet the current safety requirements in the area of image encryption. To meet the encryption efficiency and resist common attacks, we use a new space-time coupled map lattice map as the pseudo-random number generator to design a novel image encryption scheme. Moreover, compared with the traditional space-time coupling mapping, MCML produces a larger range of pseudo-random numbers, a wider range of parameters, and a good pseudo-random nature.

In recent years, many scholars have proposed many excellent algorithms for chaotic image encryption. Since the chaotic encryption schemes based on shuffling-diffusion architecture have been proposed and developed by Fridrich^2^, it have received remarkable research attention in the past decades. Due to the properties of chaotic systems, such as random-like behaviors and sensitive to initial conditions etc., they have been rapidly applied to image encryption. In the study of chaotic encryption, chaotic maps are the more important research direction. Classical one-dimension chaos, especially the Logistic map and Arnold map^3^, is usually chosen to encrypt images. Patidar *et al*.^4^ proposed a new loss-less symmetric image encryption scheme adopts substitution-diffusion architecture which based on logistic map and chaotic standard. Sam *et al*.^5^ designed a new secure algorithm for direct encryption of color images based on transformed logistic maps. Zhou *et al*.^6^ developed a new structure to construct effective chaotic systems adopting a combination of two one-dimension chaotic systems. However, the basic shortcomings of small secret key space and weak security of the one-dimensional chaotic systems limit its application, people began to turn their attention to high-dimensional chaotic system^7^. A new two-dimensional Sine ICMIC modulation map is obtained by using Sine map and iterative chaotic map with infinite collapse is generated by Liu *et al*.^8^. Wang *et al*.^9^ investigated a new hybrid color image encryption scheme which adopts two complex chaotic systems: complex Lorenz and complex Chen systems. Although high-dimensional systems have more complex dynamics behavior and better chaotic performance, but they also cost large amount of hardware resources and higher computational time overhead so that they are not suitable for real-time encryption^6^. As a kind of spatiotemporal chaotic system, coupled map lattice (CML) has both the advantages of one-dimensional and high-dimensional system, therefore, it attracted much attention in recent years. CML system represents a kind of the dynamics evolution both in time and space. It has more complex nonlinear phenomena than one-dimensional maps and lower numerical difficulty than high-dimensional chaos. Coupled map lattices are employed to generate the gray value sequences randomly to change the gray values in Wang’s image encryption scheme^10^. Besides, some improvement methods on CML system also were proposed. Zhang *et al*.^11^ proposed a novel spatiotemporal dynamics of the mixed linear-nonlinear coupled map lattices (MLNCML) and it has better cryptographic features than the logistic map or other coupled map lattices. Zhang *et al*.^12^ improved the dynamic performance of logistic map in every lattice and the CML with parameter *q* is provided with Euler method. Motivated by above discussions, we design an enhanced spatiotemporal chaos system based on CML model by applying more than one nonlinear function *f*(*x*). In this paper, we discuss the mixed couple map lattices (MCML) composed of three different chaotic maps, which are the Logistic map, Sine map and Tent map. Of course, it also can be extended to mixtures of any number of one-dimensional chaos. At the same time, we also change the coupling methods in different lattices and different nonlinear functions. Compared with one-dimensional chaos and CML system, bifurcation diagrams and Lyapunov exponents are analyzed to prove our proposed spatiotemporal model have larger range of parameters and higher Lyapunov exponents which are more suitable for the image encryption.

Traditional method of secret key generation is generally given a random bit stream. The key is independent with the plaintext so that it doesn’t have enough ability to resist common attacks^13,14^. To resist the choice of plaintext attack, many researchers take hash value of all the plaintext as the key^15,16^. But it isn’t desirable when the size of images is too large or the number of pictures is too many. It needs the long waiting time during the process of converting all plaintext into hash values. This paper introduces the key binding method that the random bit stream is disturbed by the average of all plaintext. We design a perfect key binding scheme and key distribution rule to ensure the sensibility of the encryption algorithm.

At present, most research works of the encryption schemes are committed to the improvement of scrambling process but less considered about the diffusion process. Generally, the diffusion process adopted a fixed formula by using a simple XOR operation^17–19^. As far as the diffusion process is concerned, we found that this model has a great defect^20–22^. Although we don’t know the key, we still can get the equivalent random sequence by the image attack with all pixels of 0 or 1. Therefore, we propose an improved diffusion method by converting a portion of the pixel’s values from an integer to a decimal point. At the same time, the chaotic sequences of MCML and nonlinear functions are stacked to produce a better diffusion effect by the rule we defined. The control parameter of nonlinear function is decided by decimal chaotic data and keep changing with different images. In addition, bit-level scrambling not only changes the position information, but also changes the value of the pixel, so that the bit-level based cryptosystem has higher security than pixel scrambling^23–25^. A new fast scrambling of pixel’s position scheme for Z-scan method based on bit level is applied into our algorithms to achieve higher encryption security^26,27^. The non-repeated and random sequences are produced based on comparison between the numerical value of chaotic data and Z-scan strategy have better scrambling effect than progressive-Scan.

The rest of the paper is organized as follows. Section 2 is the introduction of the proposed MCML model. Section 2.4 explain the key binding and distribution rule, the improved diffusion scheme and the bit Z-scan scrambling method. Section 3 presents the image encryption scheme using MCML system in detail. Section 4 is the experimental results, analysis and comparison. Finally, Section 5 is the conclusion of the paper.

## The new mixed couple map lattices system

### The definition of mixed couple map lattices

CML map is a typical spatiotemporal chaotic system which include some excellent advantages: more initial parameters, long periods, uneasy to be degraded and more complex nonlinear behavior, etc.^10^. Generally speaking, it considers the lattice of *L* logistic maps. It is defined as follows:

1 $${x}_{n+1}(i)=(1-\varepsilon )f[{x}_{n}(i)]+\frac{\varepsilon }{2}\{f[{x}_{n}(i-1)]+f[{x}_{n}(i+1)]\},$$

where *ε* (0 ≤ *ε* ≤ 1) represents the coupling coefficient, *i* (*i* = 1, 2, …, *L*) denotes the lattice and *f*(*x*) is the logistic map as Eq. (2):

2 $$f(x):{x}_{i+1}=4\mu {x}_{i}(1-{x}_{i}).$$

In *f*(*x*), *u* (0 ≤ *u* ≤ 1) is the parameter. When *u* ∈ [0.87, 1], *f*(*x*) is in chaos. Even if the *u* changes a little, the sequence *x* will be completely different. In addition, Sine and Tent maps are another two normally used 1-D chaotic maps. They also can be applied in the CML as the nonlinear functions. The definition can be represented by the following Eqs. (3) and (4), respectively,

3 $$g(x):{x}_{i+1}=\alpha \,\sin (\pi {x}_{i}),$$

4 $$h(x)=\{\begin{array}{rcl}{x}_{i+1} & = & 2\beta {x}_{i}.{x}_{i} < 0.5\\ {x}_{i+1} & = & 2\beta (1-{x}_{i}),{x}_{i}\ge 0.5\end{array},$$

where parameter *α* and *β* is within the range of (0, 1] and they have the same features with logistic map.

Based on the above research work, this paper proposed a novel mixed couple map lattices (MCML) by applying Logistic, Sine, Tent maps into CML model together. Three different kinds of chaos are sequentially inserted into all the lattices in MCML. The overall framework of the new MCML model is described as follows:

5 $$MCML=\{\begin{array}{l}Logistic\,lattices:{x}_{n+1}(i)=\,{\rm{mod}}(f[{x}_{n}(i)]+h[{x}_{n+1}(i-1)]+g[{x}_{n}(i+1)],1),i\,mod\,3=1\\ Sine\,lattices:{x}_{n+1}(i)=\,{\rm{mod}}(g[{x}_{n}(i)]+f[{x}_{n+1}(i-1)]+h[{x}_{n}(i+1)],1),i\,mod\,3=2\\ Tent\,lattices:{x}_{n+1}(i)=\,{\rm{mod}}(h[{x}_{n}(i)]+g[{x}_{n+1}(i-1)]+f[{x}_{n}(i+1)],1),i\,mod\,3=0.\end{array}$$

As seen in Eq. (5), we not only use three different maps to build the structure of MCML model but also change the coupling method in different lattices. Compared with CML, the coupling coefficient *ε* is disappeared and MOD operation is used in MCML map. Anything else, the data at *n* + 1 time point is only related to the data at *n* time point in CML, we also make the data at *n* + 1 point have relationship with both *n* and *n* + 1 point. To achieve better chaos, we make the parameters of three chaotic systems interact with each other. In Logistic lattices, the parameters *α*, *β* in *h*(*x*) and *g*(*x*) equal 1 − *u* in *f*(*x*). In Sine lattices, the parameters *u*, *β* in *f*(*x*) and *h*(*x*) equal 1 − *α* in *g*(*x*). In Tent lattices, the parameters *u*, *α* of *f*(*x*) and *g*(*x*) equal 1 − *β* in *h*(*x*). The details are shown as follows:

$$Logistic\{\begin{array}{l}{x}_{n+1}(i)=\,{\rm{mod}}(4\mu {x}_{n}(i)(1-{x}_{n}(i))+2(1-\mu ){x}_{n+1}(i-1)+(1-\mu )\sin (\pi {x}_{n}(i+1)),1),\,{x}_{n}(i+1) < 0.5\\ {x}_{n+1}(i)=\,{\rm{mod}}(4\mu {x}_{n}(i)(1-{x}_{n}(i))+2(1-\mu )(1-{x}_{n+1}(i-1))+(1-\mu )\sin (\pi {x}_{n}(i+1)),1),\,{x}_{n}(i+1)\ge 0.5,\end{array}$$

$$Sine\{\begin{array}{l}{x}_{n+1}(i)=\,{\rm{mod}}(\alpha \,\sin (\pi {x}_{n}(i))+4(1-\alpha ){x}_{n+1}(i-1)(1-{x}_{n+1}(i-1))+2(1-\alpha ){x}_{n}(i+1),1),{x}_{n}(i-1) < 0.5\\ {x}_{n+1}(i)=\,{\rm{mod}}(\alpha \,\sin (\pi {x}_{n}(i))+4(1-\alpha ){x}_{n+1}(i-1)(1-{x}_{n+1}(i-1))+2(1-\alpha )(1-{x}_{n}(i+1)),1),{x}_{n}(i-1)\ge 0.5,\end{array}$$

$$Tent\{\begin{array}{l}{x}_{n+1}(i)=\,{\rm{mod}}((2\beta {x}_{n}(i)+(1-\beta )\sin (\pi {x}_{n+1}(i-1))+4(1-\beta ){x}_{n}(i+1)(1-{x}_{n}(i+1)),1),{x}_{n}(i) < 0.5\\ {x}_{n+1}(i)=\,{\rm{mod}}((2\beta (1-{x}_{n}(i))+(1-\beta )\sin (\pi {x}_{n+1}(i-1))+4(1-\beta ){x}_{n}(i+1)(1-{x}_{n}(i+1)),1),{x}_{n}(i)\ge 0.5,\end{array}$$

where *u*, *α* and *β* is the control parameters and *x*_1_(*i*) = 1, 2, …, *L* are the initial values of MCML system.

### Bifurcation diagram and space-time plot analysis

**Figure 1:**
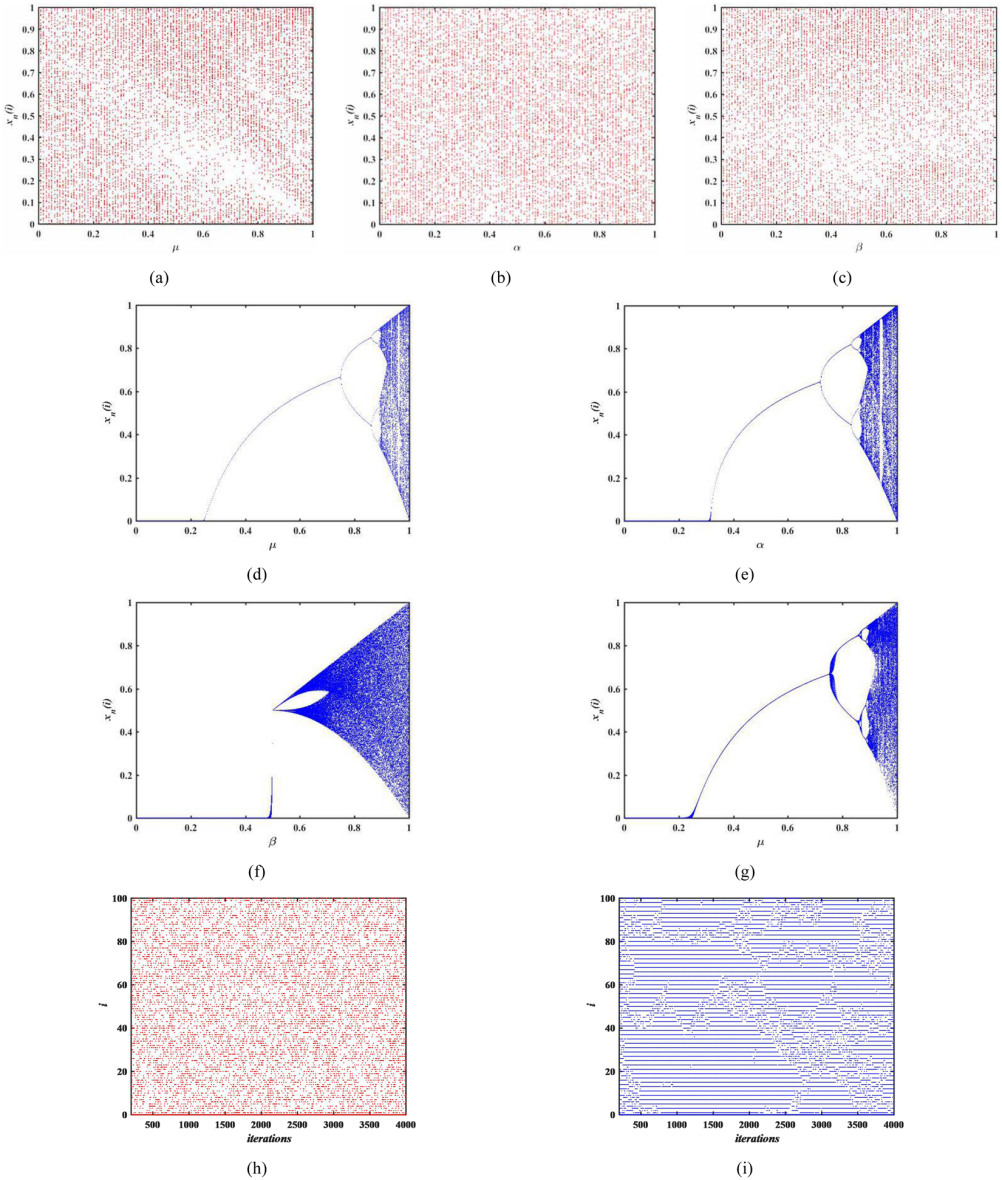
Bifurcation diagram and space-time plot analysis.

Bifurcation diagram is an important feature indicating the behavior of chaotic systems^11^. Figure 1(a–c) draw up the bifurcation of three different map lattices (Logistic Lattice, Sine Lattice and Tent Lattice) of MCML model. Figure 1(d–g) compare the bifurcation diagrams with different chaotic systems. Among them, Fig. 1(d) is the bifurcation of Logistic map, Fig. 1(e) shows the bifurcation of Sine map, Fig. 1(f) is the bifurcation of Tent map and Fig. 1(g) is the bifurcation of CML system. From these figures, we can find that the bifurcation behavior of three different map lattices of MCML and their trajectories are evenly distributed over the entire space from 0 to 1. Besides, the bifurcation diagram of the proposed MCML model without black and periodic windows are also the new features. Therefore, MCML is considered to be a spatiotemporal chaotic system suitable for cryptography.

Figure 1(h,i) show the space-time plot of MCML system and CML system respectively. Obviously, the CML system shows the defect turbulence pattern. However, the MCML system shows the fully developed turbulence pattern and the chaotic defect do not occur.

### Lyapunov exponents

**Figure 2:**
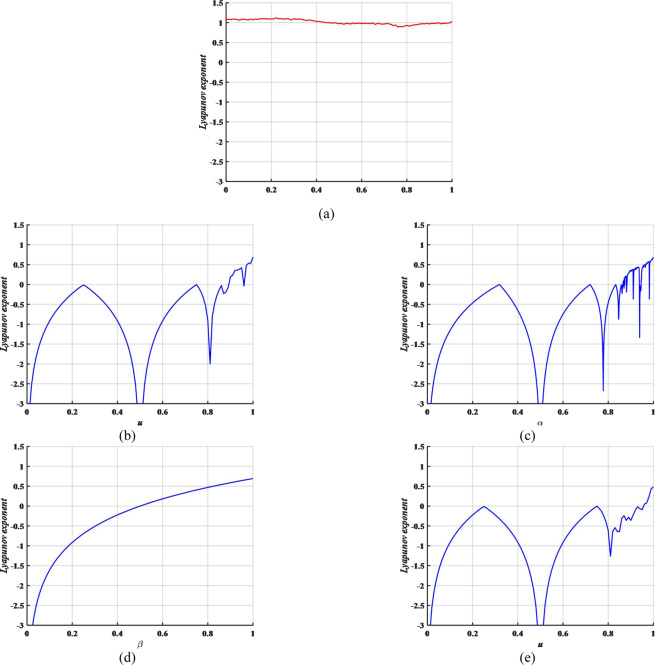
The Lyapunov exponents.

Lyapunov exponents (LE) is an important indicator for evaluating the dynamic behavior of chaotic systems and it is concerned with its predictability^8^. This paper adopts the wolf method to calculate all the LEs in every lattice of the proposed MCML and CML system. The Kolmogorov-Sinai entropy density is the average of the positive LEs of all lattices. Here, the entropy density *h* is employed to indicate whether a system is chaotic and the dynamics performance of chaos, which is described as Eq. (6).

6 $$h=\frac{{\sum }_{i=1}^{L}{\lambda }^{+}(i)}{L},$$

where *L* represents the number of lattices, ${\lambda }^{+}(i)$ indicates the positive LE of the *i*-th lattice output time series. With the fixed *α* = 0.3187, *β* = 0.2559, we consider the entropy density *h* as the LE of MCML system and do the contrast experiment between the Logistic map, Sine map, Tent map and CML system. The result is shown in Fig. 2. Obviously, MCML system possesses higher exponents than logistic map, Sine map, Tent map and CML system so that the chaotic orbits generated by MCML are much harder to predict. At the same time, it is chaos when *u* is in (0, 1). Therefore, the secret key space has increased significantly and it means higher security if MCML is applied into image encryption.

### NIST test of chaotic sequence

In order to further analyze the random characteristics of chaotic sequence generated by MCML system, the National Institute of Standards and Technology (NIST) is adopted to detect the randomness of chaotic sequence in this paper.

First, let *μ* = 0.175127105787396, *α* = 0.506205391837284, *β* = 0.630946466699243, then gives rational initial iteration value of each lattice, chaotic sequence can be gotten. In this paper, we take the number of groups M=100 and the sequence length of each group N=1000000. And then the statistical tests are performed using NIST SP 800-22 suit. NIST test consists of 15 sub-tests, all tests can be used to estimate the randomness of the sequence. The test results mainly show the pros and cons of the pseudo-random sequence by analyzing the uniformity and pass rate of the sequence, in which probability value (*P-value*) represents the uniformity of the sequence, and *Proportion* represents the pass rate of the sequence^28^. In this paper, each test gives a significance level *α=*0.01. If *P-value* ≥ *α*, the sequence is random, otherwise the sequence is not random. The results are listed in Table 1. We can clearly see that most of the *P-value* are over 0.01 and the *Proportion* are over 98% except for overlapping template test. The results of statistical tests show that the pseudo chaotic sequences generated by MCML system have good randomness.

**Table 1:** NIST test.

Sub-tests	P-Value	Proportion	Pass/Fail
Frequency Test	0.699313	99/100	Pass
Block Frequency Test (m = 128)	0.834308	100/100	Pass
Cumulative Sums Test-Forward	0.534146	99/100	Pass
Cumulative Sums Test-Reverse	0.983453	99/100	Pass
Runs Test	0.289667	98/100	Pass
Longest Run Test	0.249284	100/100	Pass
Discrete Fourier Transform Test	0.096578	99/100	Pass
Rank Test	0.071177	99/100	Pass
Non Overlapping Template Test (m = 9)	0.971699	100/100	Pass
Overlapping Template Test (m = 9)	0.883171	95/100	Pass
Universal Test	0.455937	98/100	Pass
Approximate Entropy Test (m = 10)	0.474986	100/100	Pass
Serial Test (m = 16)	0.964295	100/100	Pass
Random Excursions Test (x = −1)	0.699313	56/56	Pass
Random Excursions Variant Test (x = −1)	0.455937	53/56	Pass
Linear Complexity Test (M = 500)	0.574903	100/100	Pass

### The image encryption algorithm preliminary work

#### The key binding and distribution rule

The most of key streams are generated through the hash function with all plaintext as input and converted into one-time key as initial conditions and parameters of chaotic system^15,16^. However, it will cost a lot of calculation time. In this paper, with the average of plaintext values as interference source, the key binding and distribution rule is designed to realize a clear balance of sensitivity and time overhead. The details of rule are shown as follows:

**Step 1**. Generate a random 256-bit binary secret key stream and convert it into a 64-bit hexadecimal number: *key*’. In our simulation experiments,

$$key{\prime} ={\prime} 3{\rm{D}}5{\rm{B}}2{\rm{B}}0{\rm{B}}1{\rm{F}}946{\rm{E}}81{\rm{A}}72{\rm{C}}81{\rm{ED}}0{\rm{AE}}5{\rm{A}}770{\rm{DF}}79{\rm{F}}63{\rm{DB}}2023{\rm{EB}}26A59333{\rm{B}}44735{\rm{AB}}7{\prime} .$$

**Step 2**. Calculate the average of all plaintext pixel’s values and produce a 8-bit hexadecimal number *k’* by Eq. (7). For Pepper image of size *M* × *N*, *k*’ = ‘11E9386AE’. Select 8-bit as the finally *k*’ = ‘1E9386AE’

7 $$k{\prime} =dec2hex(floor((sum(img)/(M\times N\times 255)\times {10}^{10})).$$

**Step 3**. Use *k*’ to perturb the *key*’. Since the high bit has a greater amount of information, so the 1-th, 9-th, 17-th, 25-th, 33-th, 41-th, 49-th, 57-th of *key*’ is replaced by *k*’, then,

$$key={\prime} 1{\rm{D}}5{\rm{B}}2B0{\rm{BEF}}946{\rm{E}}81972{\rm{C}}81{\rm{ED}}3{\rm{AE}}5{\rm{A}}7708{\rm{F}}79F63{\rm{D}}62023{\rm{EB}}2{\rm{AA}}59333{\rm{BE}}4735{\rm{AB}}7{\prime} .$$

**Step 4**. In our design scheme, there are 7 initial conditions and parameters as keys: *u*, *α*, *β*, *x*_0_, *y*_0_, *z*_0_, *c*_0_. The distribution rule is given as follows:

$$u=\,{\rm{mod}}(double(hex2dec(key(1:8))/{2}^{32})+double(hex2dec(key(33:40))/{2}^{32}),1),$$

$$\begin{array}{l}\alpha =\,{\rm{mod}}(double(hex2dec(key(9:16))/{2}^{32})+double(hex2dec(key(41:48))/{2}^{32}),1),\\ \beta =\,{\rm{mod}}(double(hex2dec(key(17:24))/{2}^{32})+double(hex2dec(key(49:56))/{2}^{32}),1),\end{array}$$

$${c}_{0}=\,{\rm{mod}}(double(hex2dec(key(25:32))/{2}^{32})+double(hex2dec(key(56:64))/{2}^{32}),1),$$

$$u=\,{\rm{mod}}(double(hex2dec(key(33:40))/{2}^{32})+double(hex2dec(key(41:48))/{2}^{32}),1),$$

$$u=\,{\rm{mod}}(double(hex2dec(key(49:56))/{2}^{32})+double(hex2dec(key(57:64))/{2}^{32}),1).$$

#### The improved diffusion scheme

Most of the diffusion process is based on the simple operation as Eq. (8) and it can quickly change the values of pixels^4–6,18–20^. *p*(*i*) is plaintext, *c*(*i*) represents cipher-text, *s*(*i*) is the pseudo-random integer chaotic sequence with values between 0 and 255.

8 $$c(i)=p(i)\oplus s(i)\oplus c(i-1).$$

But, after analysis, there is a huge security risk. If all pixel’s values of an image are 0, the process of diffusion with using Eq. (8) to encryption can be described as follows:

$$\begin{array}{rcl}c(1) & = & s(1)\oplus c(0),\\ c(2) & = & s(2)\oplus s(1)\oplus c(0),\\ c(3) & = & s(3)\oplus s(2)\oplus s(1)\oplus c(0),\\ c(n) & = & s(n)\oplus s(n-1)\oplus s(2)\oplus s(1)\oplus c(0),\end{array}$$

Then, we can easily get the equivalent chaotic sequence *s*. Due to above problem, we propose an improved diffusion scheme. It is shown as follows:

9 $$\{\begin{array}{rcl}x1 & = & floor({\rm{mod}}(u\times 1000,\,256)\\ x2 & = & x2=floor({\rm{mod}}(\alpha \times 1000,256)\\ c(1) & = & \begin{array}{c}floor({\rm{mod}}(p(1)+s(1)\times {10}^{10}\\ \,+floor({10}^{10}\times 4\times (x1/255)\times (1-x1/255))+x2,\,256)\end{array}\\ c(2) & = & \begin{array}{c}floor({\rm{mod}}(p(2)+s(2)\times {10}^{10}\\ \,+floor({10}^{10}\times 4\times (x2/255)\times (1-x2/255))+c(1),\,256)\end{array}\\ c(i) & = & \begin{array}{c}floor({\rm{mod}}(p(i)+s({\rm{i}})\times {10}^{10}\\ \,+floor({10}^{10}\times 4\times (c(i-2)/255)\times (1-c(i-2)/255))+{\rm{c}}({\rm{i}}-1),\,256),\,i\ge 3\end{array}\end{array}$$

To enhance the effect of encryption, the control parameter of nonlinear function is decided by decimal chaotic data and keep changing with different images. These measures ensure that it’s hard to break the diffusion process and can’t get the equivalent chaotic sequence *s*.

#### The bit Z-scan scrambling method

**Figure 3:**
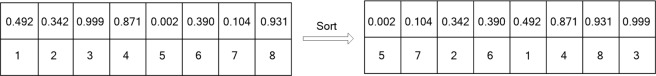
The Sort method.

In the scrambling process, the random positions of pixels or bits are generated by the MOD operation but can’t guarantee the occurrence of non-repetitive positions. Hence, A method to generate non-repeated and random data by sort the chaotic sequence is adapted. The following Fig. 3 demonstrates its principle. Suppose there is one group of chaotic sequence with size of *M* = 8, The index of the original sequence is used as the additional information of the *M*. Sorting *M*, and then the new index of the original is taken as *InM* which is the non-repeated sequence between 1 and *M*. Similarly, we can prepare sequence *InM* between 1 and *N*.

**Figure 4:**
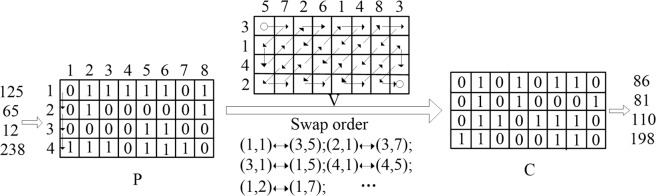
The procession of bit Z-scan scrambling method.

Suppose an image with size of 4 × 1 and convert it into bit level with size of 4 × 8. Firstly, get the *InM* = [5, 7, 2, 6, 1, 4, 8, 3], *InN* = [3, 1, 4, 2] by the above sort method to form a virtual coordinate matrix *V*. The plaintext *P* is scanned by column direction and *V* is scanned by Z-scan method. Finally, the cipher *C* is achieved through the mutual exchange of position coordinates. The process of bit Z-scan scrambling method is shown as the following Fig. 4. The result shows that our algorithm implements the function of scrambling and diffusion simultaneously.

## The new image encryption algorithm using MCML system

**Figure 5:**
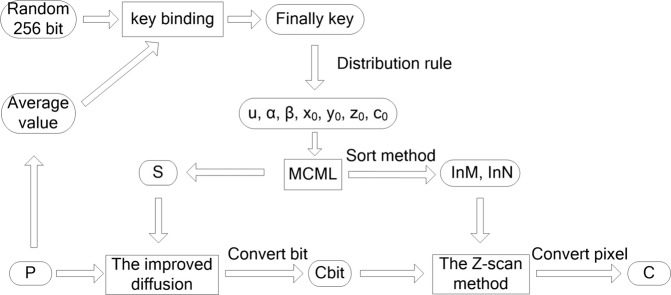
The procession of the encryption scheme.

In this section, there are several highlights in our proposed algorithm. Firstly, due to the excellent dynamic performance and larger key space, the high-dimensional chaotic MCML model is applied to generate the random sequences. Secondly, the advanced secret key binding and distribution rules are used to produce the parameters, initial values and the improved diffusion scheme is employed to enhance security against the potential attacks. Finally, the bit Z-scan method not only achieve the effect of scrambling but also diffuse the encrypted image. Figure 5 gives a brief description of the encryption scheme.

Without loss of generality, we assume that the plain image *P* sized *M* × *N*, and the lattices of MCML system is *N*. The proposed scheme can be generalized as follows.

**Step 1**. Calculate the average of the plaintext and generate a random key stream. Through the defined key binding rule, we can get the finally key.

**Step 2**. According to the rules of distribution, we can achieve the parameters of MCML system: *u* in Logistic lattices, *α* in Sine lattices, *β* in Tent lattices and *c*_0_. At the same time, we also can get the initial values: *x*_0_, *y*_0_, *z*_0_ in Logistic, Sine, Tent maps respectively. Take the *x*_0_, *y*_0_, *z*_0_ into the following Eq. 10 and iterate *N*/3 times, then, put the data into the corresponding type of lattices. Finally, we can get the initial values of *N* lattices in MCML.

10 $${x}_{i+1}=3.9999u{x}_{i}(1-{x}_{i}),$$

11 $${y}_{i+1}=0.9999\,\sin ({\rm{\pi }}{y}_{i}),$$

12 $$\{\begin{array}{rcl}{z}_{i+1} & = & 1.9999\beta {z}_{i},{z}_{i} < 0.5\\ {z}_{i+1} & = & 1.9999\beta (1-{z}_{i}),{z}_{i}\ge 0.5\end{array}$$

**Step 3**. Iterate MCML system *M* times and the chaotic matrix *S* sized *M* × *N* is constructed. Taking Eq. (9) (the improved diffusion method) to change the values of *P*, then, the diffusion image *Cbit* is achieved after converting image in bit level.

**Step 4**. Iterate MCML system to get two sequences with size of *M* and 8 × *N*. Sort the two sequences, we can get the non-repeated random sequences *InM* and *InN* respectively, then, a virtual coordinate matrix *V* is created. Take the Z-scan method which is shown in Fig. 4 to scramble and diffuse image *Cbit* in bit level.

**Step 5**. The ciphertext is converted to the pixel form and the ciphertext *C* is obtained.

**Figure 6:**
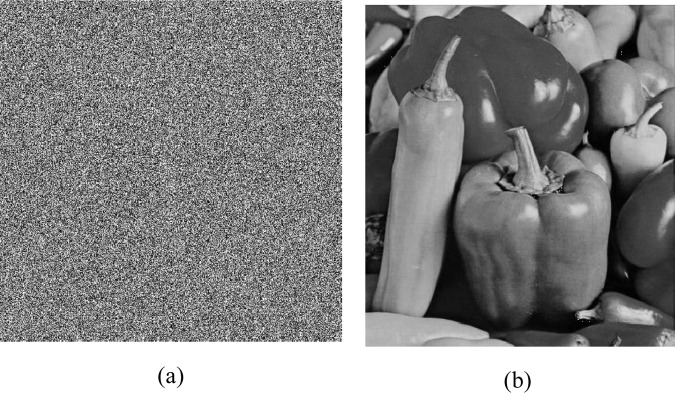
The encryption and decryption results of Pepper.

The decryption scheme is the reverse process of the encryption. Follow the diverse steps and then we can get the decrypted image. Figure 6 gives the encryption and decryption results of Pepper image. Figure 6(a,b) are the ciphertext and decoding images, respectively.

## Performance and Security Analysis

In this section, several different types of analysis measures are being used to demonstrate the security performance of the proposed image cryptosystem. Here, we show experimental results for eight different sized images. They are as follows: sized 256 × 256: Plane, Cam; sized 490 × 490: Pepper, Hill; sized 512 × 512: Baboon, Barb, Bridge, Elaine.

### Key space analysis

For an effective scheme, the secret key space should be large enough to resist-brute force attacks. From the perspective of cryptanalysis, the key space should be at least 2^100^ ^22,23^. The secret key of our introduced algorithm has a length of 256 bit, so that the key space of the encryption scheme (2^256^) is large enough to resist all kinds of brute-force attacks.

### Sensitivity analysis

#### Key sensitivity analysis

A qualified encryption algorithm should be extremely sensitive to minor changes to its secret key^24^. There are two aspects that can reflect key sensitivity: (1) a single bit change in the key should generate a totally different ciphered image, (2) if we use two decryption keys with minor differences to recover the encrypted image, the restored image should be totally different. Suppose *K*_2_ and *K*_3_ are two different keys derived from the original key *K*_1_ with one-bit change. They are given as follows:

$$\begin{array}{rcl}{K}_{1} & = & \text{'}4D5B2B0BAF946E81772C81ED1AE5A770EF79F63D32023EB27A59333B44735AB7{\prime} ,\\ {K}_{2} & = & \text{'}4D5B2B0BAF946E81772C81ED1AE5A770EF79F63D32023EB27A59333B44735AB8{\prime} ,\\ {K}_{3} & = & \text{'}4D5B2B0BAF946E81772C81ED1AE5A770EF79F63D32023EB27A59333B44735AB9{\prime} .\end{array}$$

**Figure 7:**
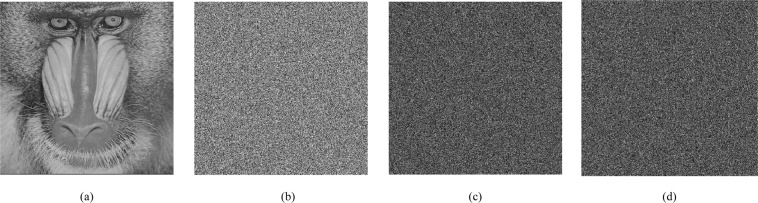
Sensitivity analysis.

The key sensitivity analysis result is shown in Fig. 7. Figure 7(a) shows the decryption Baboon with the original key *K*_1_ and Fig. 7(b) is the wrong decryption image using *K*_2_. The difference between two images which are decrypted by the wrong key *K*_2_ and *K*_3_ is given in Fig. 7(c). In addition, when using *K*_1_ and *K*_2_ to encrypt ordinary images, the encryption results are completely different, and their difference is Fig. 7(d). As shown, our algorithm is extremely sensitive to secret key in both encryption and decryption.

#### Plaintext sensitivity analysis

**Figure 8:**
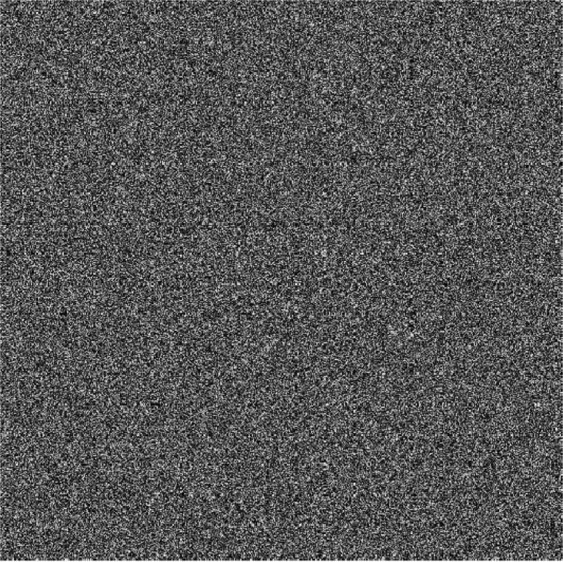
The difference of two encryption results with one pixel change.

Obviously, A qualified encryption algorithm should also be extremely sensitive to its plaintext changes^22^. We encrypt two different Baboon image whose tiny change is only one pixel. Figure 8 shows the difference between two encrypted images. It shows that the encrypted result is totally different even if two plaintexts have one-pixel change. Therefore, the proposed algorithm has high plaintext sensitivity.

### Information entropy analysis

Information entropy provides the most important qualitative criterion for image randomness^7^. Let *m* be the information source, based on Shannon’s theory, the calculation formula of information entropy is as follows:

13 $$H(m)=\mathop{\sum }\limits_{i=0}^{{2}^{M}-1}p({m}_{i}){\log }_{2}\frac{1}{p({m}_{i})}$$

where *P*(*m*_*i*_) is the occurrence probability of *m*_*i*_, *M* is the total state of the information source and the value of information source is between 0 and 255. The ideal theoretical value of information entropy of 256 gray-level images is about 8. The more closer it is to 8, the less possible an attacker can crack an image. The information entropy of ciphertexts is shown in Table 2.

**Table 2:** Information entropy of ciphertexts.

image	Plane	Cam	Pepper	Hill	Baboon	Barb	Bridge	Elaine
Information entropy	7.9972	7.9971	7.9991	7.9992	7.9993	7.9993	7.9992	7.9993

As seen in Table 1, the entropy of all ciphers is closer to 8 and it proves that the ciphertext is random dataset of pixels. Meanwhile, we also provide the contrast data with other advanced schemes which is listed in Table 3. Compared with these existing algorithms, our scheme achieves a higher information entropy so that information leakage during the encryption process is negligible, and the proposed scheme is sufficient to resist entropy attacks.

**Table 3:** The comparison in information entropies.

Encryption methods	Information entropy
ref. ^16^	7.9973
ref. ^32^	7.9975
ref. ^18^	7.9977
ref. ^19^	7.9973
ref. ^24^	7.9982
**Our algorithm**	**7.9987**

### Statistical attack analysis

#### The histogram analysis

**Figure 9:**
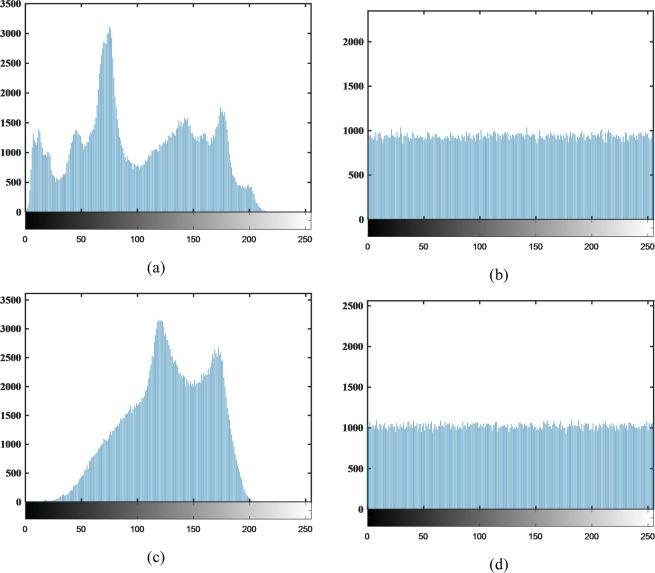
The histograms of plaintexts and ciphertexts.

The histogram of the image represents the distribution of the pixels. Generally speaking, the values for the plaintexts are concentrated in some grayscale levels, so their histogram is not uniform. To resist the potential statistical attacks, an encryption scheme should make the histogram of ciphertexts as flat as possible. The Pepper’s and Baboon’s histograms of plaintext and its histograms of ciphertext are presented in Fig. 9. As Fig. 9(b,d) shown, obviously, the histograms are very uniform, so it’s hard to reveal any useful information which indicates that attackers can’t deduce the original image by employing statistical analysis method.

#### χ^2^ test

The result of *χ*^2^ test can further analyze the distribution of pixel values in image. The value of *χ*^2^ test can be calculated as follow:

14 $${\chi }^{2}=\mathop{\sum }\limits_{i=0}^{255}\frac{{({v}_{i}-{v}_{0})}^{2}}{{v}_{0}},{v}_{0}=M\times N/256,$$

where *v*_*i*_ represents the real frequency of pixel value *i* appears and *v*_0_ means expected frequency. In this paper, the significant level α = 0.05 and ${\chi }_{0.05}^{2}=293.24783$. Results of *χ*^2^ test of plaintext images and ciphertext images are shown in Table 4. The data shows that the *χ*^2^ value of ciphertext images are all blow critical value. We can infer that the distribution of pixel value of encrypted images is uniform, which means the proposed algorithm has good ability to resist statistical attack.

**Table 4:** χ^2^ test analysis.

Image	Plane	Cam	Pepper	Hill	Baboon	Barb	Bridge	Elaine
Plain	157773	93282	216248	148041	259931	144839	144928	140650
Cipher	254	259	260	278	264	268	291	257
Pass/Fail	Pass	Pass	Pass	Pass	Pass	Pass	Pass	Pass

#### The correlation analysis between two adjacent pixels

**Figure 10:**
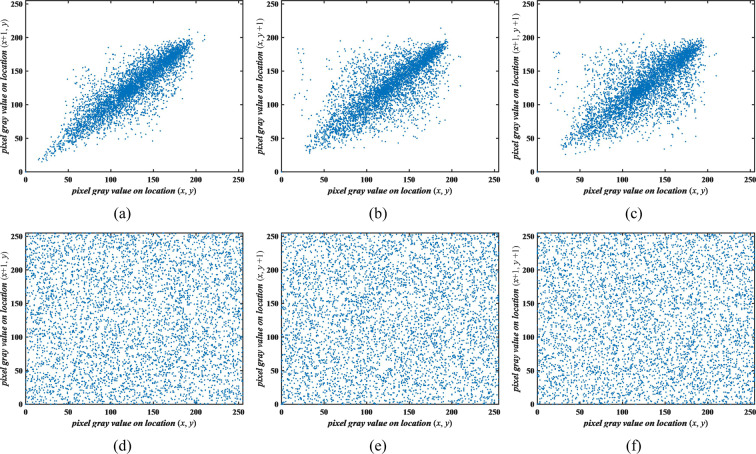
Correlation analysis.

The strong correlation between adjacent pixels is an important feature for an image and it can be applied to carry out cryptanalysis attack. Good encryption should achieve a sufficiently low correlation between adjacent pixels of a cipher image with horizontal, vertical and diagonal directions. 5000 pairs of adjacent pixels in Baboon’s cipher image are selected randomly in three directions, and their correlation is as shown in Fig. 10. As seen in Fig. 10(d–f), the proposed algorithm dramatically randomized the pixels.

In addition, calculate the correlation coefficient (*CC*) *r*_*xy*_ for each pair using the following equation:

$$cov(x,y)=E\{(x-E(x))(y-E(y))\},$$

$${\gamma }_{xy}=\frac{cov(x,y)}{\sqrt{D(x)}\sqrt{D(y)}},$$

where *x* and *y* are the pixel values of two adjacent pixels of an image,

$$E(x)=\frac{1}{N}\mathop{\sum }\limits_{i=1}^{N}{x}_{i},$$

and

$$D(x)=\frac{1}{N}\mathop{\sum }\limits_{i=1}^{N}{({x}_{i}-E(x))}^{2}.$$

Select 3000 pairs pixels in three directions to calculate the correlation coefficients in every test. The test was performed 50 times and we recorded the average of each group. The details are listed in Table 5. The measured *CC* of plaintext are close to 1 while the ciphertext are nearly 0 which indicates the correlation between adjacent pixels have been successfully eliminated by our proposed algorithm^29,30^. In addition, the contrast experimental results with the different schemes are shown in Table 3. Compared to other cryptosystems, our proposed obtains the lower correlation values in all directions and achieves a better performance in image encryption effect.

**Table 5:** Correlation analysis.

Algorithm		Plaintext			Ciphertext		
		Horizontal	Vertical	Diagonal	Horizontal	Vertical	Diagonal
Our proposed algorithm	Plane	0.9633	0.9615	0.9279	0.0013	0.0036	0.0014
	Cam	0.9745	0.9838	0.9597	0.0044	0.0016	0.0008
	Pepper	0.9876	0.9888	0.9774	0.0003	0.0009	0.0019
	Hill	0.9730	0.9745	0.9527	0.0019	0.0007	0.0019
	Baboon	0.8601	0.7541	0.7231	0.0003	0.0051	0.0025
	Barb	0.8591	0.9590	0.8414	0.0037	0.0014	0.0003
	Bridge	0.9426	0.9305	0.9031	0.0001	0.0005	0.0001
	Elaine	0.9855	0.9837	0.9742	0.0011	0.0023	0.0020
**Our Mean**					**0.0016**	**0.0020**	**0.0014**
ref. ^16^					0.0009	0.0028	0.0027
ref. ^32^					0.0033	0.0092	0.0055
ref. ^18^					0.0113	0.0173	0.0099
ref. ^19^					0.0007	0.0015	0.0014
ref. ^24^					0.0013	0.0007	0.0019

### Robustness analysis

**Figure 11:**
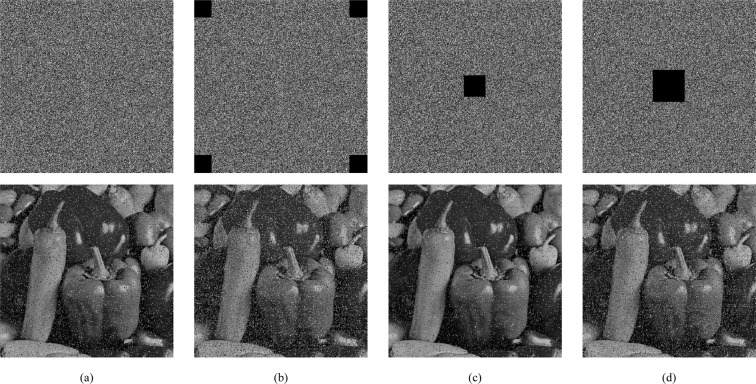
Robustness analysis.

It is easily contaminated by noise or the risk of data loss during transmission or storage over the network and physical channels^31^. An effective cryptosystem should be robust against data loss or noise interference to some extent. Randomly change pixel values of 2% (as shown in Fig. 11(a)). Figure 11(b–d) give the decryption results in different locations and numbers of data loss of ciphertext. Obviously, It is robust enough to withstand noise and data loss attacks to some extent.

### Differential attack analysis

The ability of resisting differential attack is the most important requirement for all image encryption system, and also known as chosen-plaintext attack. It is an effective way to crack the cryptosystem so that the encryption results must be different when the plaintext have a little change on a pixel. *NPCR* (number of pixels change rate) and *UACI* (unified average changing intensity) are used to evaluate the cryptographic system’s ability to resist differential attacks^19^. Mathematically, the computational formulas of the *NPCR* and *UACI* are defined as follow:

15 $$NPCR=\frac{{\sum }_{i,j}D(i,j)}{M\times N}\times 100 \% ,$$

16 $$UACI=\frac{1}{M\times N}\left(\sum _{i,j}\frac{|{C}_{1}(i,j)-{C}_{2}(i,j)|}{255}\right)\times 100 \% ,$$

where *M* × *N* are the row and column of an image respectively. If *c*_1_(*i*, *j*) ≠ *c*_2_(*i*, *j*), then *D*(*i*, *j*) = 1, otherwise, *D*(*i*, *j*) = 0. Then, A matrix *D* is created. For an image, the ideal value of *NPCR* is 99.6094% and *UACI* is 33.4635%^24^.

In our experiments, one pixel is randomly selected to add 1 to generate new original image and encrypted again. The *NPCR* and *UACI* of different images are shown in Table 6. Obviously, our scheme achieves a satisfactory performance with *NPCR* is 99.6102% and *UACI* is 33.4336% which are close to the ideal values. Therefore, our encryption scheme is very sensitive to small changes in plaintext. Table 7 is the comparison with different schemes. It’s indicated that our experimental results are similar to the results of other algorithms and the proposed scheme could effectively resist chosen plaintext attack.

**Table 6:** Differential attack analysis.

Image	Plane	Cam	Pepper	Hill	Baboon	Barb	Bridge	Elaine
NPCR (%)	99.6292	99.5819	99.6034	99.6202	99.5964	99.6395	99.6033	99.6140
UACI (%)	33.3715	33.4868	33.4402	33.4399	33.4483	33.4652	33.5469	33.4082

**Table 7:** Comparisons of differential attacks.

Algorithm	ref. ^16^	ref. ^32^	ref. ^18^	ref. ^19^	ref. ^24^	Our proposed
Mean NPCR (%)	99.6084	99.6025	99.6177	99.5842	99.6093	**99.6110**
Mean UACI (%)	33.4023	33.4937	33.6694	33.4936	33.4076	**33.4509**

### Encryption time analysis

In this paper, the proposed algorithm is implemented using software Matlab 2016a. The operation system used is Windows 7 based on x64 processor, the central processing unit (CPU) applied is Core i5-5257 2.7 GHZ and the random-access memory (RAM) adopted is 8 GB. Table 8 shows encryption time for images of different sizes.

**Table 8:** Comparisons of encryption time of 8-bit gray images for different size (in seconds).

Image size	ref. ^16^	ref. ^32^	ref. ^18^	ref. ^19^	ref. ^24^	Our proposed
128 × 128	0.29		0.052			**0.531932**
256 × 256	6.01	<0.4	0.095		0.8342	**0.668939**
512 × 512	35.59	1	0.497			**1.067064**
1024 × 1024	253.88	3	2.513			**11.587562**

## Conclusion

Finally, A new MCML system is designed by applying several simple one-dimensional maps into CML model. Furthermore, the coupling method between adjacent lattices also has been changed. The analysis results of the bifurcation diagram, Lyapunov exponents and results of NIST test demonstrate that our proposed MCML spatiotemporal chaos owns more complex dynamic behavior so that it’s more suitable for image encryption than one-dimensional or high-dimensional chaos. After, we adopt MCML system to encrypt image, combining novel strategies of key binding and distribution rules, the improved diffusion scheme and the Z-scan scrambling method. Several different types of analysis are being used, including key space analysis, sensitivity analysis, information entropy, statistical attacks, and differential attacks. Simulation results show that our scheme has excellent encryption performance.
